# Reply to “Medical Education and Artificial Intelligence: Some Suggestions”

**DOI:** 10.31662/jmaj.2025-0384

**Published:** 2025-12-12

**Authors:** Izuki Amano, Kisho Obi-Nagata, Ayane Ninomiya, Yuki Fujiwara, Noriyuki Koibuchi

**Affiliations:** 1 Department of Integrative Physiology, Gunma University Graduate School of Medicine, Maebashi, Japan

**Keywords:** medical education, generative AI, artificial intelligence

## Dear Editor,

We sincerely appreciate Dr. Matsubara’s valuable comments and constructive suggestions ^(1)^.

As noted, our study involved a limited cohort, and we fully acknowledge the constraints in generalizing its findings ^(2)^. Outcomes could vary depending on study design, location, and timing. Another thing he pointed out, one factor underlying negative impressions on generative artificial intelligence (AI) among some students, was the burden to put considerable effort into verifying AI-generated answers, which may have surprised many readers.

We intentionally designed some questions that could induce AI errors to encourage students to compare and critically assess AI versus their own responses. This “spot-the-mistake” approach aimed to foster critical thinking, an essential skill in medical education. Your observation accurately reflects this intent, and we strongly agree with him that this aspect could be recognized as a potential teaching tool.

ChatGPT (Chat Generative Pre-trained Transformer, OpenAI) has advanced markedly from version 3.5 to 4 ^(3)^, which may challenge us to construct questions that reliably produce AI errors. As suggested, exoring quizzes where students tend to error while AI provides accurate answers is a promising future direction.

Finally, we would like to clarify our stance on AI use in medical reasoning and writing. We may not agree that AI should be completely banned, but rather that its use should be moderately regulated. In educational settings, AI should serve as a supplementary tool while learners are encouraged to identify problems, think critically, and reach conclusions independently. In clinical practice, physicians must still be able to collect, interpret, and articulate findings on their own; cultivating these fundamental abilities is essential for safely and effectively integrating AI into medical care.

We recognize that generative AI can be a powerful learning aid, valuable for organizing thoughts, guiding study directions, and brainstorming. Nonetheless, we hope our students will not only consume AI-generated information but also contribute to the advancement of AI by providing new evidence and data. At the same time, we support the use of generative AI to improve efficiency and streamline tasks in clinical practice. This trend cannot be stopped, nor should it be resisted, as it represents a natural extension of technological progress that has historically enhanced medical workflows.

Once again, we are deeply grateful for his insightful comments, which will greatly inform and improve our future research and educational efforts.

## Article Information

### Author Contributions

Izuki Amano: Wrote the manuscript. Kisho Obi-Nagata, Ayane Ninomiya, Yuki Fujiwara, Noriyuki Koibuchi: Review and editing of the manuscript.

### Conflicts of Interest

None

### Approval by Institutional Review Board

Not applicable
